# AMP-CapsNet: a multi-view feature fusion approach for antimicrobial peptide prediction using capsule networks

**DOI:** 10.1186/s44342-026-00067-6

**Published:** 2026-02-07

**Authors:** Ali Ghulam, Mujeebu Rehman, Huma Fida, Pei-Yu Zhao, Ramsha Noroze, Ye-Chen Qi, Xiao-Long Yu

**Affiliations:** 1 Information Technology Centre, Sindh Agriculture University, Tandojam, Sindh 70060 Pakistan; 2 School of Information and Communication Engineering, Guilin University of Electronic Technology, Guilin, China; 3 Center for Informational Biology, School of Life Science and Technology, University of Electronic Science and Technology of China, Chengdu, 611731 China; 4 School of Computer Science and Technology, Hainan University, Haikou, 570228 China; 5 School of Materials Science and Engineering, Hainan University, Haikou, 570228 China

**Keywords:** AMP-CapsNet, AAC, DPC, Drug discovery, Antimicrobial peptides

## Abstract

Antimicrobial peptides (AMPs) are universally found in both intracellular and extracellular settings and have significant antibiotic-resistant bacteria are becoming a bigger problem. In medical laboratories, it has shown notable anti-bacterial effectiveness in treating diabetic foot infections and related issues. New medication development frequently targets (AMPs), which are certainly ensuing components of adaptive immune system. The findings of this research employs deep learning to identify antibiotic activity. Numerous computational methods have been established to detect antimicrobial peptides via deep learning algorithms. We introduced a novel deep learning approach called antimicrobial peptides using Capsule Neural Network (AMP-CapsNet) to precisely forecast them and evaluated its efficacy against deep learning and baseline models. AMPs prediction using capsule neural networks, a type of next generation neural network, to build prediction models. Additionally, we utilized Amino Acid Composition (AAC) for effective features encoded method and as well as dipeptide composition (DPC). Every model underwent independent cross-validation and external testing. The findings indicate that the enhanced AMP-CapsNet deep learning model surpassed its counterparts, achieving an accuracy of 97.29% and an AUC score of 98.91% on the test set using with dipeptide Composition (DPC). The proposed AMP-CapsNet demonstrates superior performance of the testing set achieved accuracy 97.29% score with DPC and accuracy 84.42% score with AAC approach. Consequently, the technique we advocate is anticipated to enhance the accuracy of antimicrobial peptide predictions in the future. By producing powerful peptides for medication development and application, this study advances deep learning-based AMP drug discovery approaches. This finding has important ramifications for how biological data is processed and how pharmacology is calculated.

## Introduction

A number of recent studies have focused on discovery of novel antibiotics, including peptides, is a protracted process that may span several years and entail substantial expenses. Nonetheless, AI has recently expedited scientific discoveries, revolutionizing this domain [1]. Their primary method of action involves damaging microbial cell membranes, which leads to cell death [2]. In recent years, researchers have become increasingly interested in antibiotic resistance is a global concern that could lead to a pandemic in the future. A number of recent studies have addressed the issue of antimicrobial peptides (AMPs) prediction is the development of next-generation antimicrobials. Some naturally occurring antimicrobial peptides (AMPs) are already being used in clinical settings, making them useful templates. Predicting novel AMPs from the sequenced genomes of various organisms is a promising method to speed up the discovery of new antibiotics [3, 4]. This makes them promising candidates for use in the fields of biology, food preservation, antimicrobial cosmetics, and environmental protection [5–7]. Antimicrobial peptides are topical anti-infective drugs used in medicine to treat and prevent hospital-acquired infections as well as infections of the skin and soft tissues [8–10]. In this research author used data compelling evidence refuting the idea that (2DCNN) and supplementary features were extracted from DDE (PSSM) profiles, indicating that clathrin proteins may be identified using next gen sequencing level. [11]. Antimicrobial peptides have increasingly garnered interest in clinical therapy as viable alternative antibiotic therapies, showing promising efficacy in wound healing, infectious illness management, and anticancer therapy [12]. Simultaneously, certain studies have indicated that antimicrobial peptides can modulate endothelial cell function, facilitate angiogenesis, and enhance blood circulation in the feet, consequently benefiting the vascular lesions associated with diabetic feet [13]. Numerous studies have investigated traditional approaches to antimicrobial peptide screening make use of biological techniques and molecular dynamics models. Methods like the agar diffusion test and the lowest inhibitory concentration assessment are commonly used in biochemical techniques to evaluate the antibacterial activity of peptide segments extracted from biological sources [14, 15]. A large variety of creatures, including plants, insects, and animals, contain AMPs, a diverse set of tiny peptides with biological activity that range in length from ten to one hundred amino acids. After cecropins were found in the 1980 s, researchers began to study AMPs [16]. Because the cell membrane is also amphipathic, it is easier for AMPs to interact with the lipid bilayer of the cells being targeted, since they often have both hydrophilic and hydrophobic properties, making them amphipathic. Their antibacterial efficacy is greatly influenced by the connection between Deep-ProBind and the BERT methods [17]. Bacteria can acquire resistance to AMPs to a lesser extent than other antibiotics, although this is mostly due to the fact that AMPs' toxicity is typically caused by non-specific processes rather than targeting specific proteins [18].

Nonetheless, the advancement and evaluation of antimicrobial peptides presently encounter numerous obstacles. Initially, conventional biochemical techniques are expensive and entail protracted development timelines. The simulation process may be limited by computational resources, leading to diminished accuracy of outcomes. Furthermore, the range of screened antimicrobial peptides may be restricted, and their stability can be inadequate to fulfill actual application standards. Therefore, the establishment of an effective, accurate, and convenient screening technique is essential. This strategy aims to address the shortcomings of existing screening methods, improve efficiency and accuracy, and lower research and development expenses. Recent studies have explored the discovery of small functional peptides is attracting an increasing amount of attention from researchers due to the fast advancements in AI and computing capacity. These little peptides typically contain anything from five to fifty amino acid residues, albeit their sequences are shortened [18]. Ample evidence [19, 20] exists essential functions in biological systems are provided by these short peptides, which include antiviral, immunoregulatory, cellular signal transduction, and antibacterial roles Machine learning techniques that have been fine-tuned can make functional peptide recognition and prediction more accurate and efficient, which will increase our consideration [21, 22] by adding constructed, multi-fusion Deep Neural Network (DNN), and structural properties. In their investigation, S Khan et al. utilized deep learning approaches to improve the finding of sumoylation peptides [23]. To date [24], no study has looked specifically utilizing machine learning and ensemble learning methodologies, Manavalan et al. were able to increase the prediction accuracy of cell-penetrating proteins and their engulfment efficacy.

There is limited research investigating the forecast DPs exclusively from protein sequences, this research details the creation of a new deep learning model, DPI CDF [25]. The suggested DPI_CDF model is an example of a hierarchical deep forest that makes use of all three of these encoding methods. Consequently, the limitations of wet lab tests are mitigated by computational methods to precisely anticipate the functional kinds of DPs. Considerable research attention has been introduced an innovative multi-class ensemble prediction model, StackDPPred, designed for identifying the properties of DPs [26]. A multifaceted recognition of characteristics model utilizing a capsule neural network (MDCapsUbi) was developed to forecast protein ubiquitination locations [27]. This study presents a flexible architecture utilizing a capsule network capable of precisely identifying promoter combinations in raw DNA data of five distinct organisms, both eukaryotic and prokaryotic. Our architecture, CapsProm, facilitates the development of models with minimal effort for the task of promoter identification across various datasets [28]. The CapsNet surpassed the initial convolutional neural network design MusiteDeep and other established tools in most instances, yielding encouraging results for practical applications, particularly in scenarios involving little training data. The length of the capsule provides a precise approximation of the confidence in the PTM forecast. Therefore, rapid and accurate prediction of the ACPs by in silico approach is highly required with huge amount of peptide sequences collected in the post-genomic era. [29]. This paper introduces a new ABP recognition method, termed ABPCaps. It incorporates (CNN), (LSTM) network, and an innovative neural network known as the capsule network. The capsule network autonomously extracts essential properties using positive and negative samples, resulting in enhanced performance [30]. These methods may experience diminished computing efficiency and increased time expenditures while processing large-scale datasets. Although current methods have substantially aided in peptide identification, there is a necessity for more adaptable ways that can swiftly respond to varied identification demands. Moreover, the generalization capacity of certain models on novel datasets requires enhancement. Consequently, our research introduces a novel methodology that fills this void by creating a predictive model that provides adaptability and efficacy in detecting antimicrobial peptides across various situations. However, identifying new AMPs is often slow, laborious and expensive in wet-lab experimentation.

**Fig. 1:**
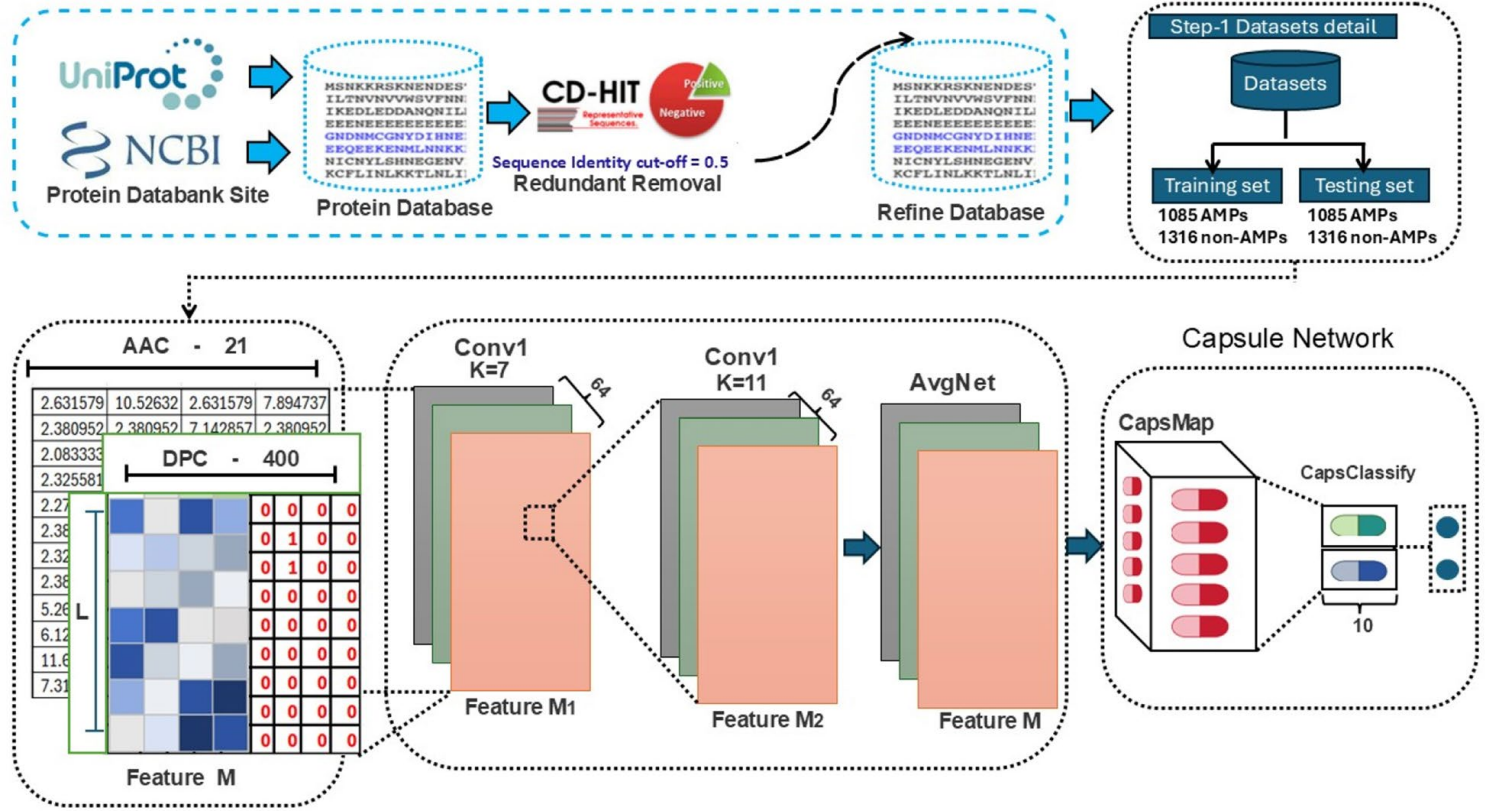
Proposed framework

This study is to develop a method for prediction of antimicrobial peptides using cutting-edge deep learning algorithms that is both accurate and efficient. Few attempts have been made to investigate the based on our trained datasets with five-fold cross-validation. We compared three different machine learning models and presented the enhanced performance of AMP-CapsNet using deep learning model. To date, scant attention has been paid to be more precise, our model derives full features from peptide sequences by using feature fusion methods, which incorporate multiple feature types. To gather properties of peptide sequences at various scales, a multi-scale convolutional layer is used. Remarkably few studies have been designed to improve the model's prediction efficacy for finding antimicrobial peptides, the improvements aim to boost its capacity to identify different features inside peptide sequences. We employed LightGBM, XGBoost, and AdaBoost as comparative machine learning models. Relatively little is understood not only did AMP-CapsNet make the cut, but so did a number of other cutting-edge, innovative deep learning models. On top of that, we compared the six groups mentioned earlier with three baseline machine learning models. To date, scant attention has been paid an external dataset was used to test the models' performance. As shown in Fig. 1, the exact steps of this research are detailed here.

## Materials and methods

### Data collection

Data on several variables were used to for screening and processing steps were finished. After the screening was finished, we obtained 1085 AMPs and 58,761 non-AMPs samples of antimicrobial peptides from the appropriate databases. The data provide convincing evidence showing that Total 59,761 data points length were utilized in this research and the datasets benchmark existing researcher utilized at [31]. We searched the UniProt database for the necessary number of peptide sequences and referred to previous studies [31] to ensure that the number of negative samples was equal to that of the antimicrobial peptide samples. Our findings can be compared to data of earlier studies that the non-positive samples were mostly composed of peptides that do not have antibacterial properties. Incorporating additional factors into their selection allowed us to keep our positive samples in a state of balance. To ensure that length was not a distinguishing factor, we made sure that these samples were the same length as our positive samples. The data provide preliminary evidence to suggest that AMP-1085 and non- AMP-1316, now contain peptide sequences. To train the model and validate it internally (using five-fold cross-validation), we used the in our proposed method experimental total 2401 dataset. To test the model's generalizability, we used the 2401 dataset, which was tested externally. After the datasets were finished, we made sure there weren't any duplicate peptide sequences in either dataset. The reliability of training and evaluating models is guaranteed by this method. We used 1,085 positive AMP sequences and 1,316 non-AMP sequences. Antimicrobial peptide prediction benchmarks are compromised by the addition of 1,316 non-AMP negative data points [32]. Benchmarks here refer to the datasets that represent a standard for assessing and comparing the performance of different computational pipelines in the scope of antimicrobial peptide (AMP) predictions. These benchmarks usually consist of pooled labeled AMP (positive) and non-AMP (negative) sequences, which are employed as training and test sets for machine learning models.

### Peptide sequence feature representation

In this study, we used a variety of models to their fullest capacity in order to identify antimicrobial peptides. We picked several feature extraction methods to match the unique needs of every single model type according to their characteristics. Few studies have investigated the impact of extract characteristics from protein sequences, we created two features extraction methods, a method that is both effective and realistically applicable. We employed the AAC and DPC feature extraction method for deep learning models. This DPC approach successfully captures the local characteristics of sequences, hence improved the model performance. Empirical evidence appears to confirm the notion that our proposed method performance was outstanding with DPC features extractions method.

### Amino acid composition (AAC)

For the purpose of this AAC is a commonly utilized feature extraction technique in bioinformatics, primarily for representing protein sequences. The twenty amino acids that comprise a protein sequence are collectively known as its Amino Acid Composition (AAC). It reveals crucial details regarding the protein's structure and functional characteristics. This section provides detailed information on characteristics is generated by combining local and global features of amino acid sequences using this approach. A widely accepted hypothesis can express any peptide sequence in the way shown in Eq. 1, and the calculation formula ***Px*** for different subscripts is given in Eq. 2. Predictive models often include amino acid composition, which refers to the frequency of each amino acid residue within a protein sequence. Let ***fx*** represent the occurrence frequencies of n's 20 native amino acids. The composition of amino acids ***Px*** is estimated using [33].

1 $$Px(n) = \frac{f_{x} (n)}{\sum\nolimits_{i=1}^{20} f_{i}(n)}\quad i, x = 1, 2,\,\ldots\!,20$$

The composition of protein *n* is thus defined as:

2 $$P(n) = [p_{1} (n), p_{2} (n),\, \ldots\!, p_{20} (n)]$$

Section [N] provides important contextual information regarding calculated the likelihood of the occurrence of the 20 natural amino acids (i.e., "ACDEFGHIKLMNPQRSTVWY") in protein sequences or peptide chains using the encoded amino acid composition. One parts, the first of which deals with the basic features used to define the structure and functions of proteins is their amino acid composition, or AAC [34]. A number of considered the implications of unexpected to see that classifier performance did not differ significantly when employing AAC characteristics or domains. Surprisingly, across most datasets and classifiers, both AAC characteristics performed as well as domains. This suggests that AAC alone captures a lot of the information needed to identify interacting proteins. This is a simple and very effective way to describe protein classification tasks. It gives you a fixed-length vector that includes the basic and chemical properties of a protein sequence. AAC provides a brief summary of protein composition, which indirectly shows physicochemical properties like polarity and hydrophobicity. It has been successfully employed in the prediction of antimicrobial peptides (AMPs), the classification of enzyme functions, and the determination of protein subcellular localization, due to its ability to distinguish between functionally diverse protein families.

### Dipeptide Composition (DPC)

It is generally agreed that the DPC: Dipeptide composition describes the kinds and amounts of every conceivable pair of amino acids that can come after each other in a peptide. The number of potential dipeptides is 400, with each one containing two amino acids in a predetermined sequence [35, 36]. The following equation can be used to represent DPC:

3 $$DPC (i) = \frac{N (i)}{N_{total}}$$

It is generally agreed that protein sequence can be better understood with the use of DPC, an approach to feature extraction. DPC Features extraction method turned their attention to better understand the connection between adjacent amino acids, it looks at the binary representation of pairs of amino acids separated by k intervals in the sequence. Empirical evidence appears to confirm the notion that the binary matrix with dimensions of 20 × 20, which corresponds to the sequence length decreased by k, is generated by the BPF method once the value of k is established. Current research seems to indicate that the sequence's amino acid pairing frequency is used to generate the matrix.

DPC incorporates local sequence order information by analyzing the interactions among adjacent residues. The feature space has a lot of dimensions (400), which could cause problems if it isn't properly regularized. It only looks at short-range pairwise linkages and doesn't take long-range dependencies into account. When used with Capsule Neural Networks, which can learn hierarchical spatial relationships, DPC characteristics let the model find both local shapes and higher-order interactive structures. This shows how well CapsuleNet-DPC works, with nearly perfect classification.

### The process of feature fusion

In this proposed method following normalization, the AAC and DPC features scale were horizontally concatenated, creating a fused vector with a dimension of 420 (20 + 400). The CapsuleNet architecture received the hybrid feature vector.

### Architectural design

#### Enhancing capsule neural network (AMP-CapsNet)

A considerable proposed method of research has focused on Capsule Neural Network module which contained on deep learning model. Current proposed hypothesize that the AMP-CapsNet module is made up of three parts: the SEM module, the MD-FRM module, and the Capsule Neural Network module. SEM module divides the unprocessed protein sequence into smaller pieces and then encodes each of those amino acids. Numerical vectors are used to depict protein fragments. Using convolutional operations and a channel attention approach, MD-FRM module is able to detect multi-dimensional concealed features. To make it easier to categorize AMP-1085 and non- AMP-1316, locations, CapsNet module combines and improves the features of two dimensions. Figure 1 shows the proposed CapsNet framework.

Current theories hypothesize that peptide feature recognition, a specific type of convolution block, is implemented to operate on the feature matrix ***M***. Current research seems to indicate that two one-dimensional convolution layers make up the convolution block, which implements linear activation functions (ReLU). We subsequently acquire the feature maps M1 and M2.

### Capsule network module (CapsNet)

The AMPs and non-AMPs sample for this study consisted of a novel deep learning method. The capsule network differs from CNN in that it uses capsules instead of scalar neurons. Each sampling unit consisted of vectors, which are collections of neurons, are represented as capsules. Similar to the architecture used in [27] the CapsMap and CapsClassify layers make up the capsule network that is covered in this article.

The present study employed a CapsMap is able to decipher intricate features because it takes features found in the character of proteins and descriptor map dimensions, integrates and refines them even further, and then turns them into capsule vectors. This proves that characteristics with a coarser grain are refined into features with a finer texture. The only difference between this layer and a one-dimensional convolution one is that capsule vectors are used instead of scalar neurons. There are just two capsule vectors in CapsClassify: one positive and one negative. Depending on their length, we can tell if a site is ubiquitinated or not.

The CapsMap used in this study may be capable of resolving complex features; it extracts features from both the sequence and feature map dimensionalities, refines them, and converts them into capsule vectors. This shows that denser features are derived from denser grains. The difference between this layer and a one-dimensional convolution one is that scalar neurons are replaced with capsule vectors. CapsClassify only has 2 capsule vectors; a positive and a negative. We can say a site is ubiquitinated or not based on their length.

The setting of the research design was capsules in CapsNM with a 10D dimension. A nonlinear map can be constructed between CapsMap and CapsClassify by the iterative approach. As described in [37], this type of nonlinear mapping makes it possible to update the internal parameters of the network and is called the dynamic routing mechanism.

When the class is not present, it penalizes capsule activations that are excessively high. To make sure that class-specific capsules activate strongly for the right class and suppress activation for the wrong classes, Capsule networks use this margin loss. An essential part of the CapsNet model, this loss function takes the magnitudes of capsule vectors into account instead of merely scalar probabilities, which helps to improve class separation. We conducted in-depth experiment based on CNNs to lose map data when they pool jobs [38, 39]. The dynamic route method in the capsule network keeps all the information that links the capsules in CapsMap and CapsClassify, which makes it better than older methods. We examined that the given hot vector, a 1D Convolutional Neural Network (1D CNN) with three hidden layers is made to pull out its buried local features. When there isn't a lot of data for one hot vector, the locally linked convolution layers turn it into a number of feature maps that show hidden structural details in the raw protein sequence.

### Model evaluation

This study used qualitative evaluation Criteria for evaluation. There was a slight difference in median values across to measure how well AMP-CapsNet worked, researchers used the Area Under the Curve (AUC) statistic. The ROC curve is plotted to check for the AUC value to see if these findings would imply that the true positive rate is shown on the vertical axis, and the false positive rate is on the horizontal axis of the ROC curve. AUC: As a main metric measure of the performance of classifiers. An interesting side finding was that we created ROC curves and used measures like Accuracy, Recall, Specificity, Precision, F1-Score, and AUC value to assess the model's performance. Our findings are consistent with previous results showing True positives (TP), true negatives (TN), false positives (FP), and false negatives (FN) are the primary evaluation metrics in the confusion matrix. Accuracy measures how many samples were correctly predicted by the model, Precision evaluates how many samples were actually positive out of all the positive ones, and Specificity shows how many true negative samples were accurately predicted [40, 41]. Overall, these studies provide support for the validity of recall and precision is represented by the F1-score. The area under the curve (AUC) measures the classifier's overall effectiveness, whereas the ROC curve shows how well it performs over all possible classification thresholds. Models with AUC values close to 1 perform exceptionally well.

## Results

We suggested a AMP-CapsNet for the prediction of protein AMPs modification sites. The AMP-CapsNet surpassed the baseline convolutional neural network design MusiteDeep and other established tools in most instances, yielding promising outcomes for practical applications, particularly in scenarios involving little training data. The length of the capsule provides a precise estimation of the confidence in the AMPs forecast. We additionally established that the characteristics of the internal capsule may be trained to function as a theme detector for AMPs sites in the absence of AMPs labels. Our research elucidates the recognition mechanism of AMPs modifications and the implications of CapsNet in different bioinformatics challenges.

### Capsule neural network (CapsNet) model results

A further complication for the present hypothesis is that achieved best performance metrics score based on our proposed method AMP-CapsNet. Table 1 demonstrates that AMP-CapsNet attained the highest performance. AMPs-DPC is more effective than AMPs-AAC. The CapsuleNet model achieves far better accuracy using AMPs-DPC (97.29% vs. 84.42%). Moreover, the differences between AMPs-AAC (sensitivity of 76.20% and specificity of 90.49%) and AMPs-DPC (sensitivity of 96.80% and specificity of 97.70%) are large. The MCC value of AMPs-DPC is 94.54, much greater than the MCC value of AMPs-AAC (68.58%) that shows a strong association between predictions and actual classes. In categorization, AMPs-DPC gives better performance than AMPs-AAC as sequence correlations are more accurately encoded in AMPs-DPC. It appears to be that the DPC encoding best suits Capsule Networks to model sequence dependencies. On the whole, the aforementioned table indicates that Dipeptide Composition (DPC) implemented with CapsuleNet effectively performs better than Amino Acid Composition (AAC) in AMP classification. The fact that this much improvement is only achieved by recording the dipeptide connections testifies that this particular representation greatly improves CapsNet in discriminating between AMP and non-AMP sequences. The proposed AMP-CapsNet demonstrates superior performance of the training set achieved accuracy 88.95% score with DPC and accuracy 81.42% score with AAC as shown in Table 1.

**Table 1:** Proposed method CapsuleNet performance metrics score based on Training Datasets

Training Datasets					
Features Encoder Method	**Methods**	**Acc (%)**	**Sn (%)**	**Sp (%)**	**MCC**
AMPs-AAC	AMP-CapsNet	81.42	79.20	88.49	61.58
AMPs-DPC	AMP-CapsNet	88.95	86.25	91.66	78.03

The proposed AMP-CapsNet demonstrates superior performance of the testing set achieved accuracy 97.29% score with DPC and accuracy 84.42% score with AAC as shown in Table 2.

**Table 2:** Proposed method CapsuleNet performance metrics score based on Testing Datasets

Testing Datasets					
Features Encoder Method	**Methods**	**Acc (%)**	**Sn (%)**	**Sp (%)**	**MCC**
AMPs-AAC	AMP-CapsNet	84.42	76.20	90.49	68.58
AMPs-DPC	AMP-CapsNet	**97.29**	**96.80**	**97.70**	**94.54**

### Capsule neural network (CapsNet) model results of ROC (AUC curve)

A possible interpretation of this finding is that the proposed AMP-CapsNet demonstrates superior performance in both the training set and outside influences test set. Comparative summary of two feature encoding approaches (i.e., AAC and DPC) to predict Antimicrobial Peptides (AMPs) using Capsule Network (AMP-CapsNet). Below, this is a description of the results as well as their theoretical implications: Calculates the frequency of each of the 20 standard amino acids into a peptide sequence AMP activity is often defined by a sequence motif or spatial arrangements, such as hydrophobic and hydrophilic patterns. It establishes local seqence order and pairwise relations, holding more context information compared to AAC. Dipeptides better mimic structural and functional characteristics (e.g., α-helices, β-sheets) critical for the action of antimicrobial peptides. This new feature space could be used by CapsuleNet to encode spatial hierarchies, which is one of the attractive properties of capsule networks. Good with detailed and structured data (like DPC) but not with simple features (AAC). Its dynamic routing system was presumably more capable of effectively representing dipeptide-based spatial hierarchies. Absolutely kills with rich, structured data (DPC) but just fails with far too basic of features (AAC). Its dynamic routing approach likely better summarizes spatial hierarchies dipeptide-based information. Hybrid encodings (AAC + DPC + physicochemical attributes) evaluation. Test CapsuleNet with other models (like CNNs, transformers) to show it is better for sequence-based tasks. Check if better capsule architectures boost performance even more with DPC. The proposed AMP-CapsNet demonstrates superior performance of the training set achieved F1 97.02% score with DPC and F1 80.63% score with AAC as shown in Table 3. We computed ROC(AUC) score based on fivefold validation ROC(AUC) 98.95% score with DPC based with training datasets and ROC(AUC) 96.05% score with AMPs-AAC same based with training datasets.

**Table 3:** Proposed method CapsuleNet performance metrics score with training sets

Testing Datasets					
Features Encoder Method	**Methods**	**F1 (%)**	**Prec (%)**	**Rec (%)**	**ACU**
AMPs-AAC	CapsuleNet	79.61	83.10	76.39	96.05
AMPs-DPC	**CapsuleNet**	**97.02**	**97.25**	**96.80**	**98.95**

According to test set for CapsuleNet with other models (like CNNs, transformers) to show it is better for sequence-based tasks. Check if better capsule architectures boost performance even more with DPC. The proposed AMP-CapsNet demonstrates superior performance of the testing set achieved F1 **97.02**% score with DPC and F1 79.61% score with AAC as shown in Table 4. We computed ROC(AUC) score based on fivefold validation ROC(AUC) **91.49**% score with DPC and ROC(AUC) 95.53% score with AMPs-AAC.

**Table 4:** Proposed method CapsuleNet performance metrics score with testing sets

Testing Datasets					
Features Encoder Method	**Methods**	**F1 (%)**	**Prec (%)**	**Rec (%)**	**ACU**
AMPs-AAC	CapsuleNet	80.63	86.85	76.20	95.53
AMPs-DPC	**CapsuleNet**	**88.65**	**91.18**	**86.95**	**91.49**

### Validation accuracy and validation lose using AMPs-DPC

**Fig. 2:**
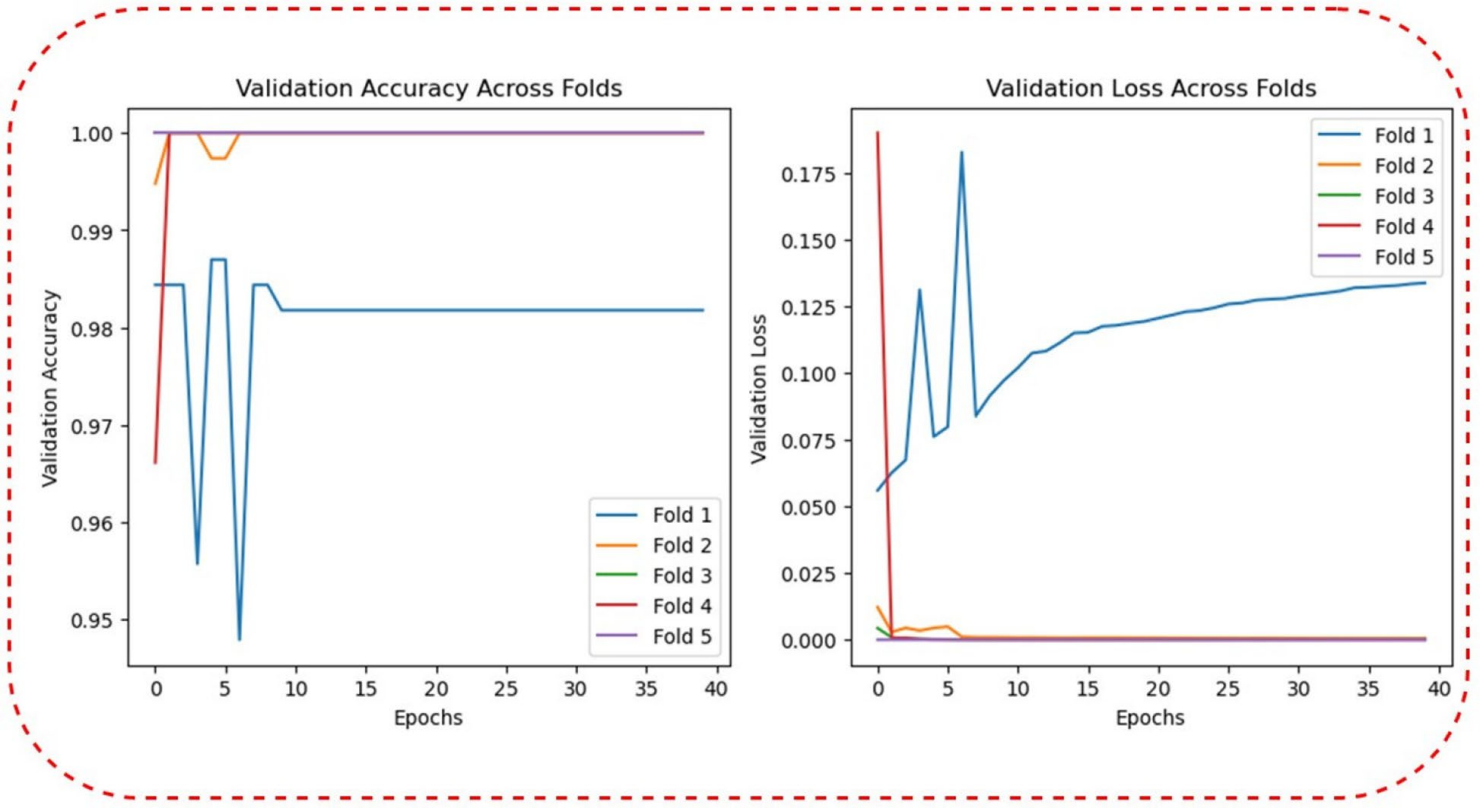
Training Set Validation Accuracy and Validation Lose using AMPs-DPC with CapsNet

Few studies have investigated the impact of AMP-CapsNet model that was proposed obtained an exceptional training accuracy of 98.09% and a validation accuracy of 98.91%. In our proposed model's performance was assessed in our study using a combination of AMP-CapsNet validation accuracy and validation lose score of 4.76% using AMPs-DPC. Figure 2. AMPs train and validation lose for sequence processing curves of accuracy. The goal of this work was to use deep learning models to enhance the prediction of protein secondary structures. Two metrics validation accuracy and validation lose were used to assess the performance of three models. The results are shown below, and their relevance is then discussed.

**Fig. 3:**
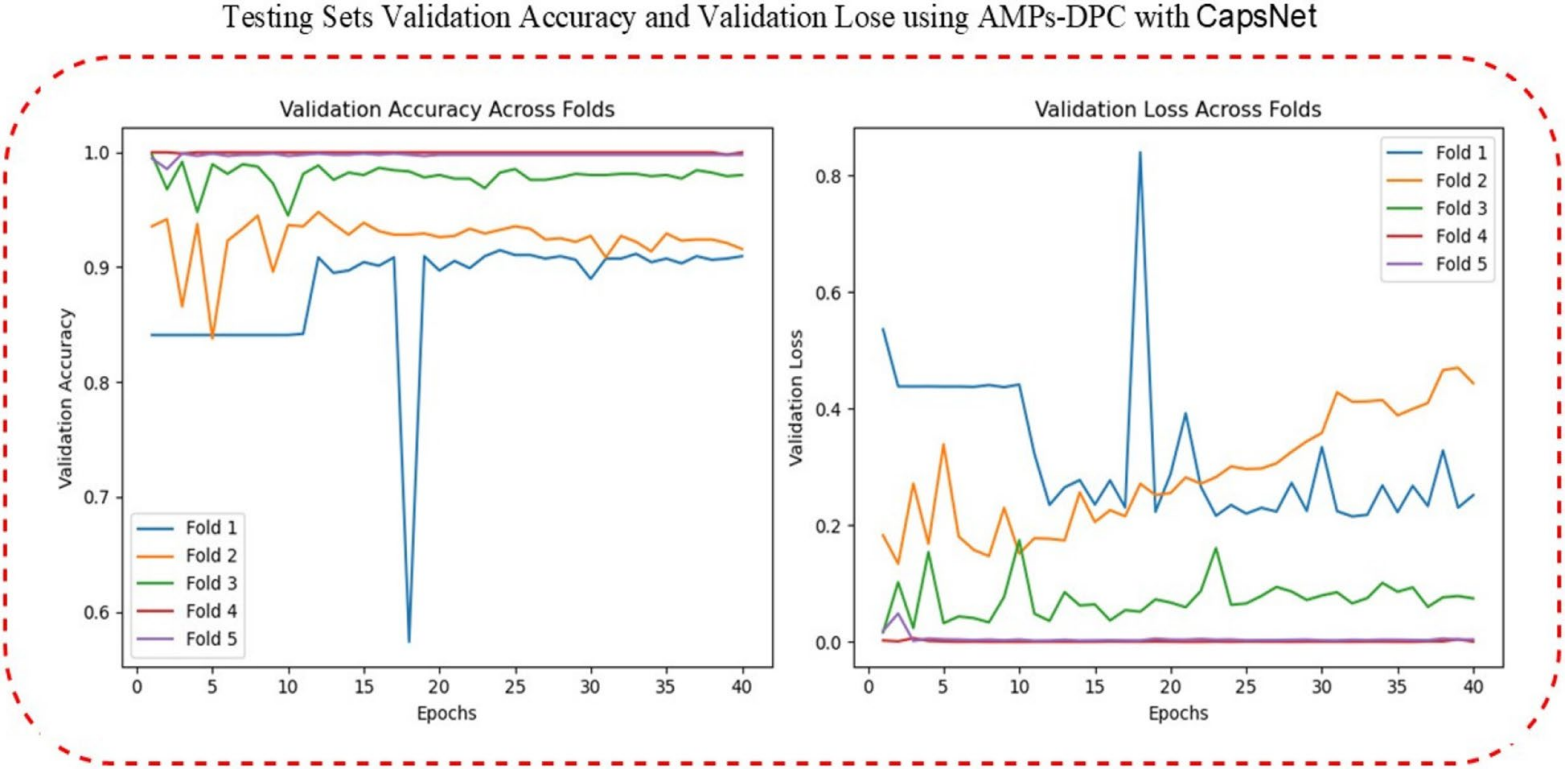
Testing Sets Validation Accuracy and Validation Lose using AMPs-DPC with CapsNet

These include implementation performance on Dipeptide Composition (DPC) based AMP datasets with capsules, shown here. The graph shows two primary evaluation metrics—validation accuracy and validation loss—on the test set as training epochs proceed. This elevated validation accuracy reflects the effectiveness of the model in correctly classifying AMPs, while the reduced validation loss indicates better generalization and lower error during model evaluation. These results demonstrate the ability of the CapsNet architecture to learn complex sequential patterns from DPC-encoded AMP data as shown in Fig. 3.

### CapsNet comparison with other three DL models for Accuracy and ROC (AUC)

**Fig. 4:**
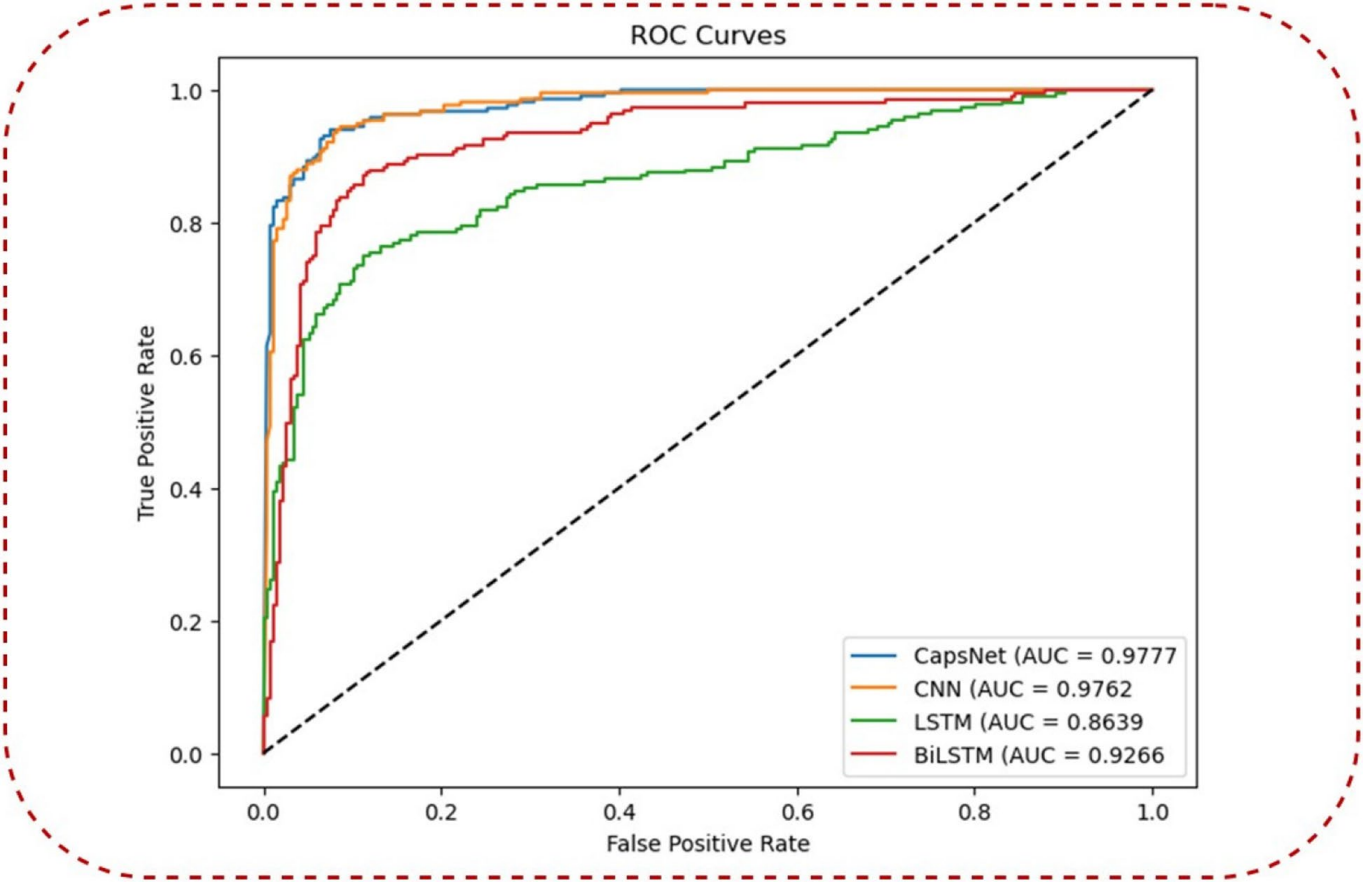
Training sets Comparison with other three DL models for Accuracy and ROC (AUC)

It is the third-best classifier with ROC (AUC) on the DPC, and the AMP-CapsNet predictor is trained on the most comprehensive database of protein AMPs Analysis. Specifically, the AUC-ROC curve is used to evaluate binary classification models, with a special emphasis on performance across thresholds, particularly in datasets that are imbalanced. The area under the receiver operating characteristic curve (AUC-ROC) is a single scalar metric that simplifies the process of comparing several models, regardless of the classification thresholds that each model uses. Due to the fact that it is not dependent on any particular threshold, it is an excellent option for conducting a fair comparison across models that have various optimal thresholds. In the proposed method CapsNet model achieved ROC (auc = 0.9777) score, CNN model achieved ROC (auc = 0.9762), LSTM model achieved ROC (auc = 0.8639) and BiLSTM model achieved ROC (auc = 0.9266) as shown in Fig. 4. One of the things that we wanted to express was that, in theory, we were going to establish a threshold, above which every result would be a 1, and below which every result would be a 0. This would imply that at the extremes, you would obtain the original condition, in which you had all 0 s and all 1 s (at a cutoff of 0 and 1 respectively), but you would also get a sequence of intermediate states that lie within the 1 × 1 graph that contains your ROC.

### Accuracy and ROC (AUC) Comparison using AMPs-DPC

**Fig. 5:**
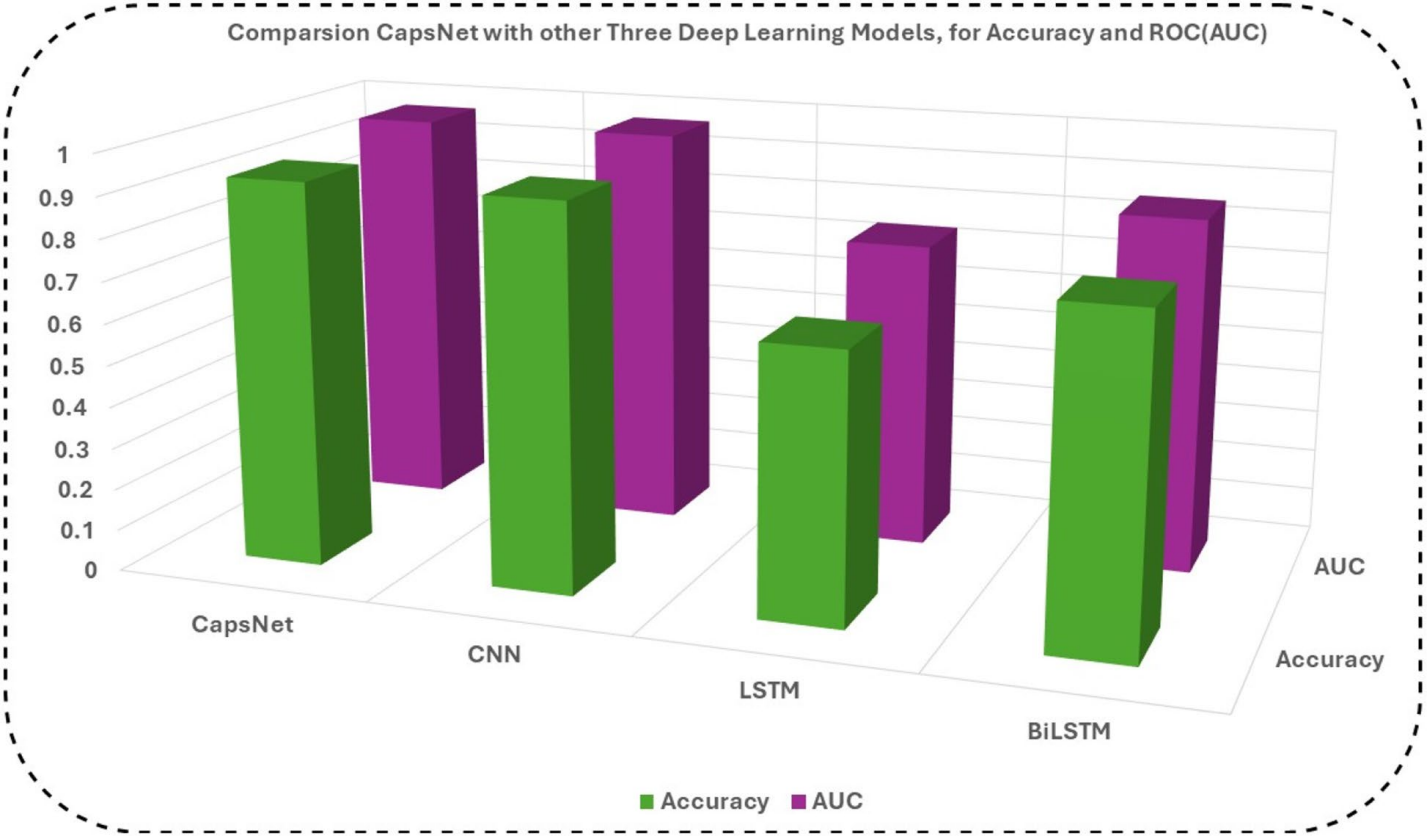
Comparison three DL models for Accuracy and ROC (AUC) using AMPs-DPC

The area under the ROC curve (AUC) is a metric that is used to measure the performance of a marker. A higher AUC value implies that the marker is performing more effectively. A diseased individual is also equivalent to the chance of having a greater marker value than a healthy individual, which is also equal to the area under the curve (AUC). In the proposed method CapsNet model achieved ROC (auc = 0.9777) score, CNN model achieved ROC (auc = 0.9762), LSTM model achieved ROC (auc = 0.8639) and BiLSTM model achieved ROC (auc = 0.9266) as shown in Fig. 5.

### Comparison results of ROC (AUC curve) using AMPs-AAC

**Fig. 6:**
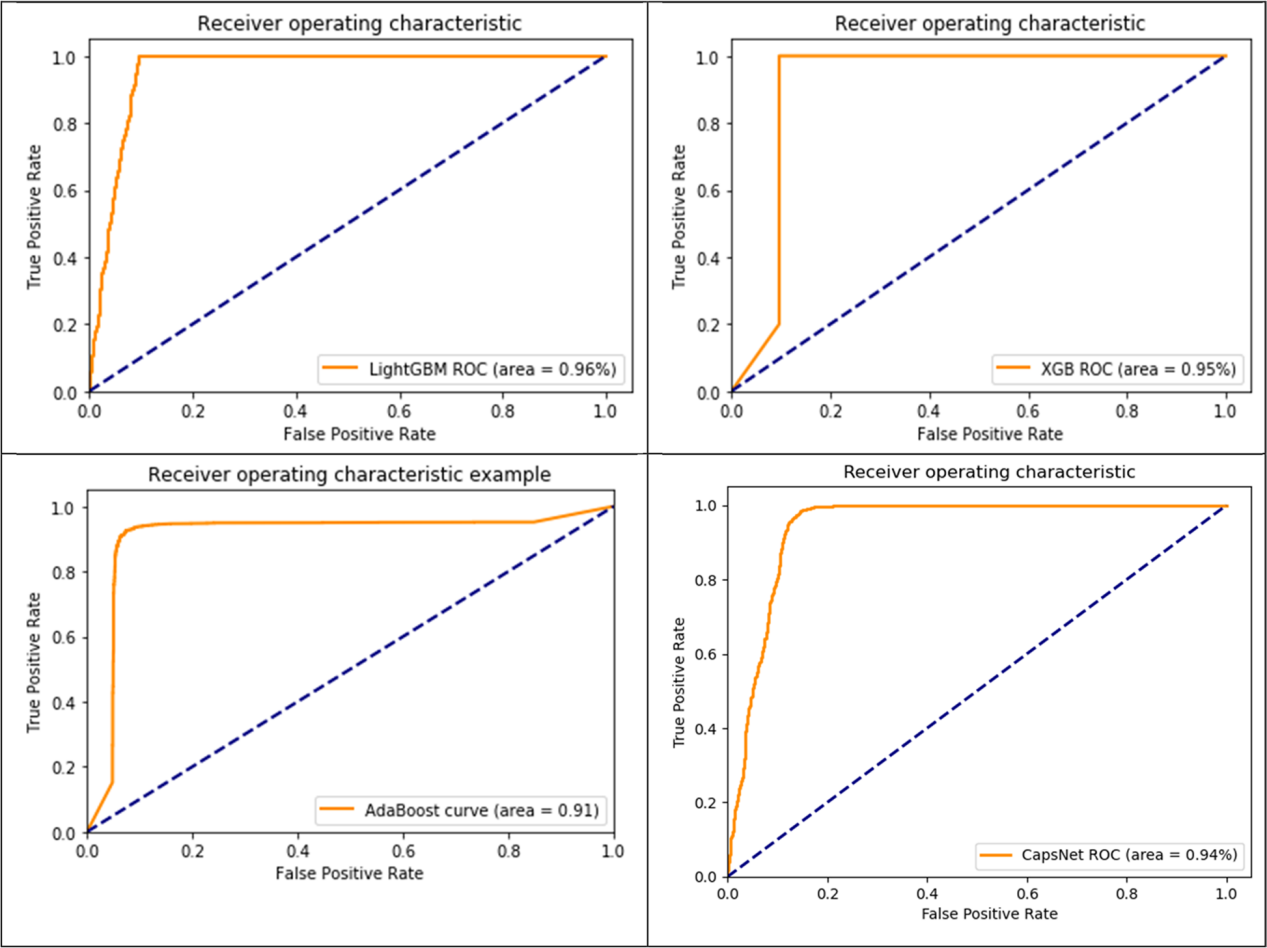
Results of Received Operating Characteristic and precision-recall curves contrasting the suggested deep architecture with two additional platforms for predicting protein AMP sites

A possible interpretation of this finding is that the proposed AMP-CapsNet demonstrates superior performance in both the training set and outside influences test set. A further complication for the present hypothesis is that in Fig. 6, we visualized the ROC and precision-recall curves, together with AUC and mean precision, for the two tools and our model. We used the predicted scores from AMPs and non-AMPs for this. we obtained 1085 AMPs and 58,776 non-AMPs samples of antimicrobial peptides from the appropriate databases. A possible interpretation of this finding is that the most instances, our deep architecture outperformed other tools in terms of AUC and mean precision, and it performed at a greater sensitivity under most specificities (as shown in Fig. 5). It proved that our design is quite reliable when used to data on protein ubiquitylation sites on a broad scale. Our findings suggest a need for greater to apply boosting classification, it trained twelve sub-models using twelve subgroups of positive training data and twelve negative samples. The goal of these classification models was to find possible homologous protein fragments that were very similar to the positive training data by focusing on the feature patterns of those samples. With a recall of less than 3.89%, it outperformed our deep architecture in terms of precision. However, when looking at ROC and precision-recall curves, our deep architecture clearly has the upper hand.

### Comparison results of ROC (AUC curve) using AMPs-DPC

**Fig. 7:**
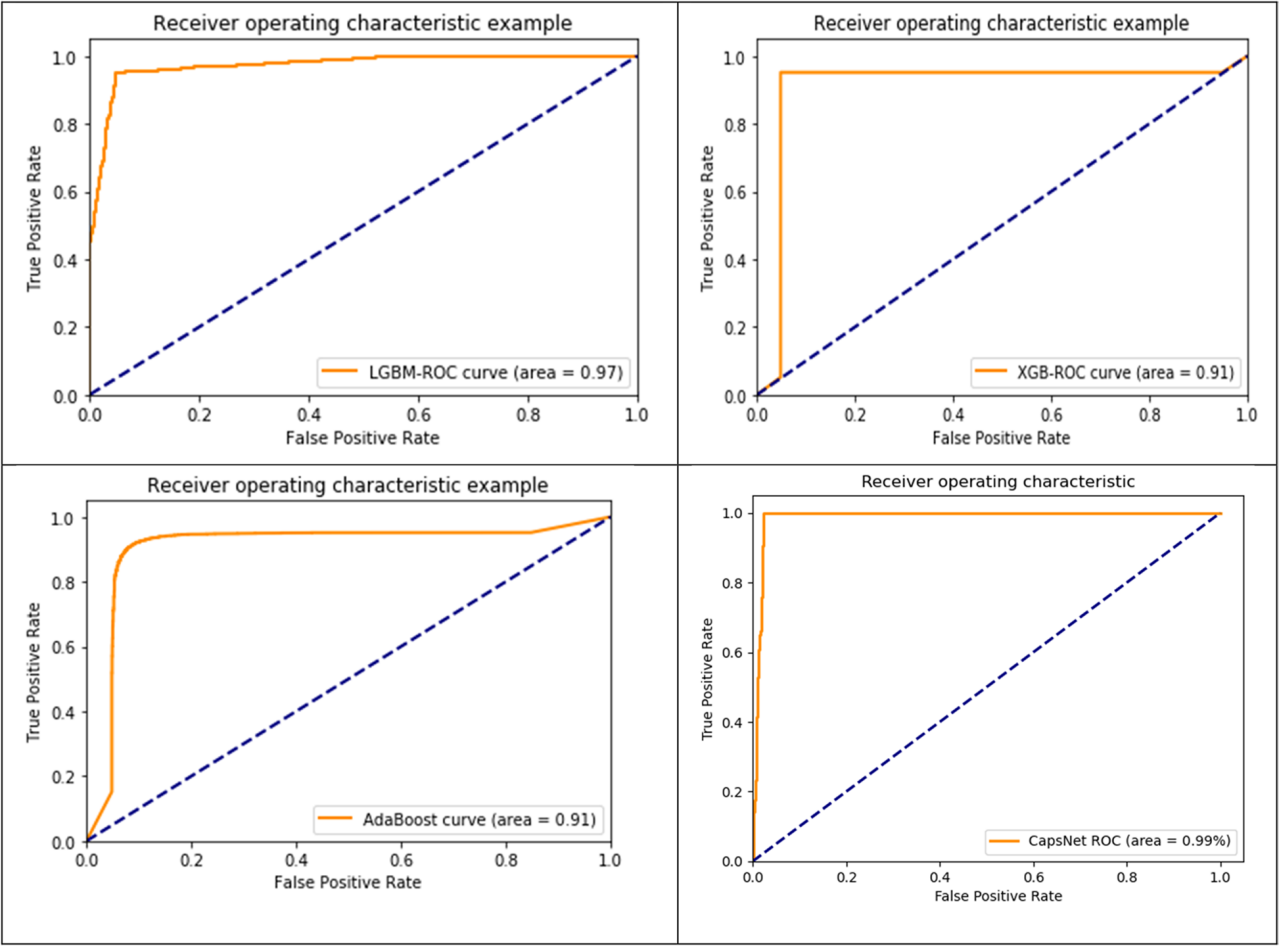
Results of Received Operating Characteristic and precision-recall curves contrasting the suggested deep architecture with two additional platforms for predicting protein AMP sites

Table 2 and Fig. 7 show that our models performed somewhat worse on the external dataset AMP-1085, despite the fact that they performed quite well on the non- AMP-1316, dataset (as shown Table 2 and Fig. 7. The data provide preliminary evidence to suggest that AMP-1085 and non- AMP-1316, now contain peptide sequences. To train the model and validate it internally (using five-fold cross-validation), we used the in our proposed method experimental total 2401 dataset. Differences in complexity, data distribution, and the amount and kind of noise in the datasets are the root cause of this problem. A model is said to have overfit when it takes in too much information from the training data, leading to overspecialization and poor performance on new, unknown data.

### CapsNet comparison Results of ROC with three ML Classifier using AMPs-AAC

**Fig. 8:**
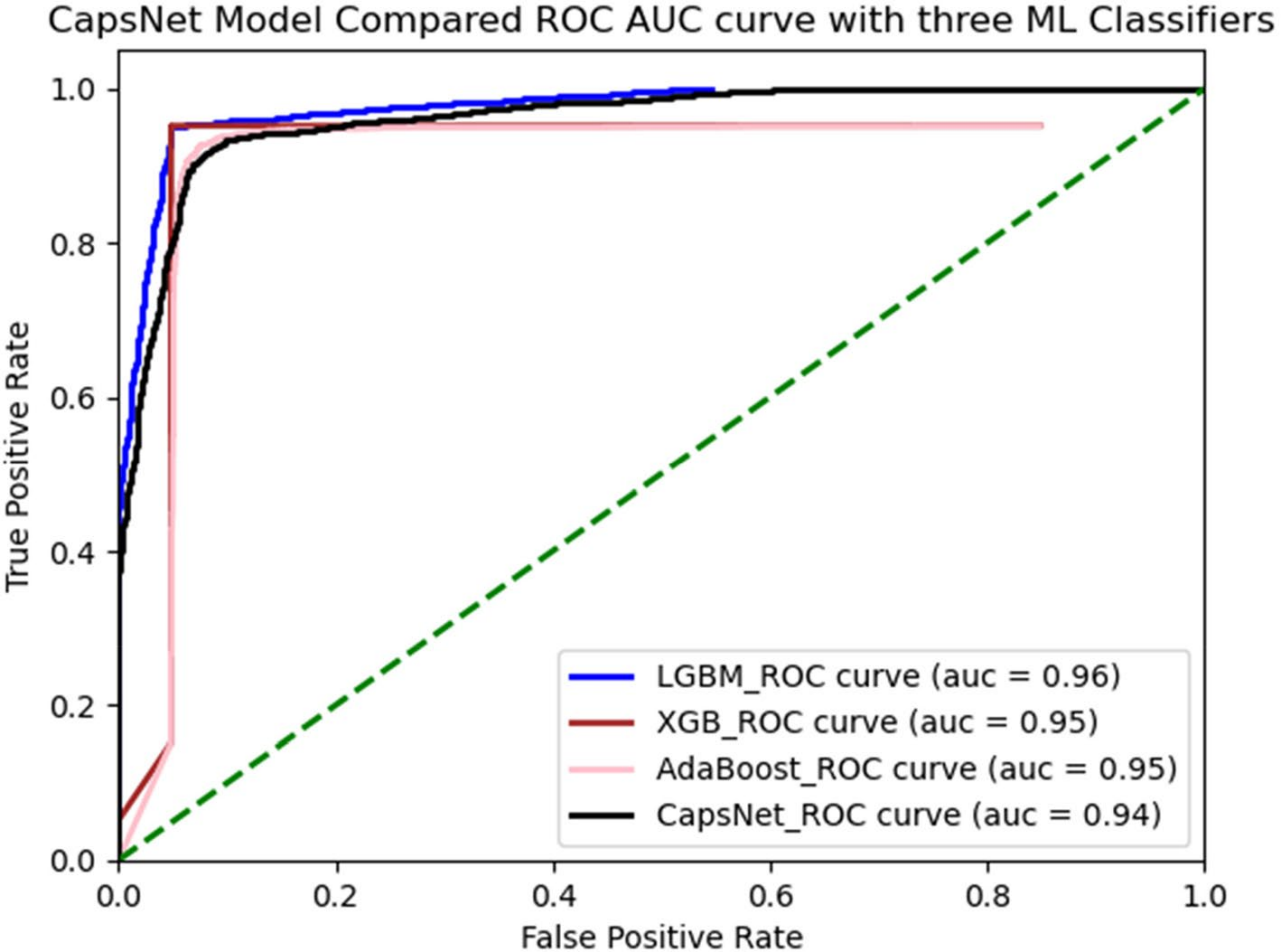
ROC with three ML classifier using AMPs-AAC

All three inputs (one-hot vectors, physicochemical properties, and PSSM profile) were trained simultaneously on the multi-modal network. The evaluation of the fully trained network and its subnets was carried out on an independent testing set. This became even more evident when you look at the proposed method LGBM classifiers recorded a ROC(AUC) SCORE 0.96%,XGB classifiers ROC(AUC) SCORE 0.95% AdaBoost classifiers ROC(AUC) SCORE 0.95% and CapsNet ROC(AUC) SCORE 0.94% So as you can see seems similar. Figure 8. Their generative ROC (receiver operating characteristic) curves and precision-recall curves.

### CapsNet comparison results of ROC with three ML classifier using AMPs-DPC

**Fig. 9:**
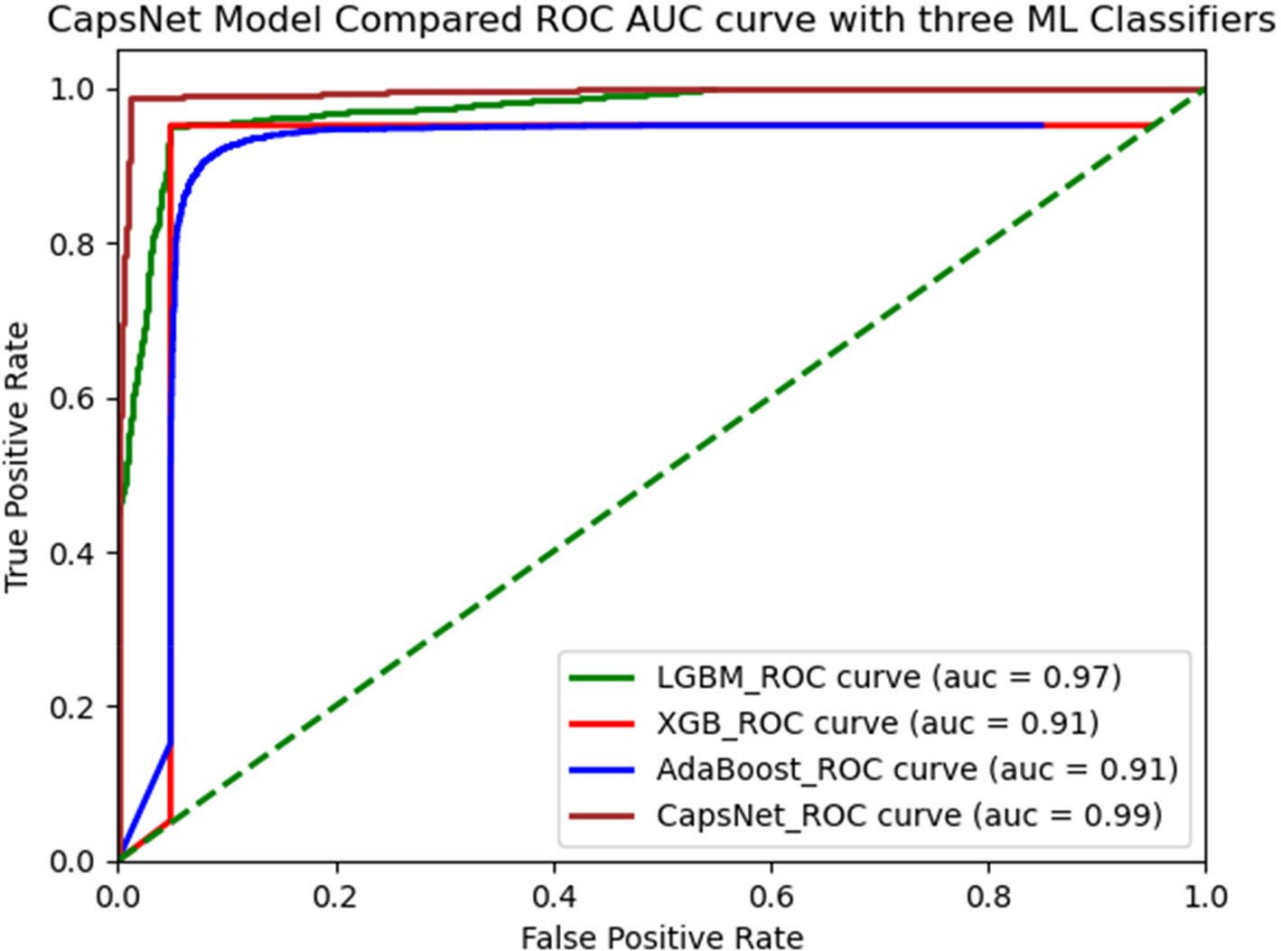
ROC with three ML Classifier using AMPs-DPC

The entire multi-modal network was concurrently trained using one-hot vectors, physico-chemical properties, and PSSM profile inputs. The fully trained network and its subnets were evaluated on an independent testing set. In order to proposed method LGBM classifiers received ROC(AUC) SCORE 0.96%, XGB classifiers received ROC(AUC) SCORE 0.95%, AdaBoost classifiers received ROC(AUC) SCORE 0.95%, and CapsNet received ROC(AUC) SCORE 0.94%, as looks like similar. Their generative ROC (receiver operating characteristic) curves and precision-recall curves are illustrated in Fig. 9.

Our findings suggest the multi-modal network was simultaneously trained utilizing one-hot vectors, physicochemical characteristics, and PSSM profile inputs. The comprehensive network and its subnetworks were assessed using an independent testing dataset. In order to proposed method LGBM classifiers received ROC(AUC) SCORE 0.97%, XGB classifiers received ROC(AUC) SCORE 0.91%, AdaBoost classifiers received ROC(AUC) SCORE 0.91%, and CapsNet received ROC(AUC) SCORE 0.99%, which is better performance then others. Their generative ROC (receiver operating characteristic) curves and precision-recall curves are illustrated in Fig. 9.

### Comparison results of performance metrics based on CapsNet

**Fig. 10:**
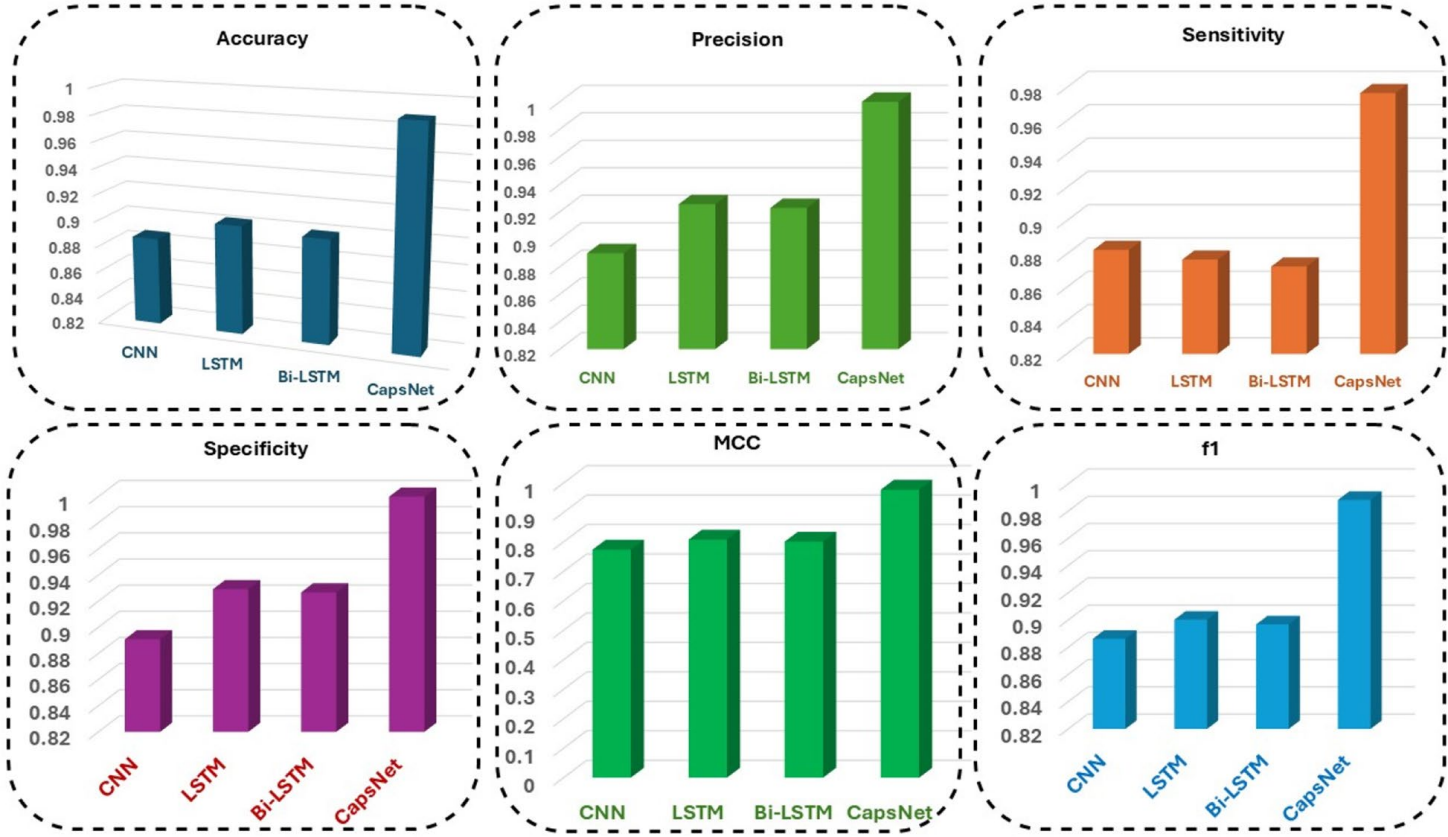
Comparison results of performance metrics based on CapsNet

This shows a comparison between deep learning models: CNN, LSTM, Bi-LSTM, CapsNet based on different performance metrics, which include Accuracy, Precision, Sensitivity, Specificity, MCC (Matthews Correlation Coefficient), and F1 score as shown in Fig. 10. Here is a summary of this metric: This metric measures the model's overall accuracy by considering true positive and true negative samples. CapsNet outperforms the other models and provides the highest accuracy followed by Bi-LSTM, LSTM and CNN in that order. Precision measures the percentage of true positive scenarios out of all positive scenarios that the model predicted. It is evident from the bar chart that CapsNet has the highest precision followed by Bi-LSTM, LSTM, and CNN. A high accuracy number represents fewer false positives. Sensitivity (known as Recall or True Positive Rate) evaluates how well the model identifies positive cases. CapsNet has the highest sensitivity which indicates its high ability to correctly classify positive samples. CNN, LSTM and Bi-LSTM have lower values as compared to CapsNet. Specificity evaluates the ability of the model to correctly predict negative cases (aka True Negative Rate). The CapsNet model exhibits the highest specificity, reflecting its ability for accurate identification of negative samples. The specificity of all models are just like their order by scores: CNN has the lowest one. 2- Matthews Correlation Coefficient (MCC) MCC is a balanced metric that takes into account all four categories of the confusion matrix — True Positives (TP), True Negatives (TN), False Positives (FP) and False Negatives (FN). High MCC values indicate a strong link between predictions and target labeling. As shown in the chart, CapsNet scored the highest MCC score and is followed by Bi-LSTM, LSTM and CNN. The F1-score is the harmonic mean of Precision and Recall which balances false positive and false negative. CapsNet is specifically built to perform well on this domain, demonstrating impressive performance on classification tasks. The F1-scores of Bi-LSTM, LSTM, and CNN are slightly lower but show a stable trend. The improved generalization capability and robustness of CapsNet is demonstrated by its consistently superior performance over CNN, LSTM, and Bi-LSTM on all the metrics. CNN outperforms only the simplest baseline, and thus highlights its bad performance in sequential dependencies when compared to LSTM models. LSTM and Bi-LSTM have decent accuracy which shows that they work well on sequence based tasks but they are not as good as CapsNet in this scenario.

### SHAP feature analysis

**Fig. 11:**
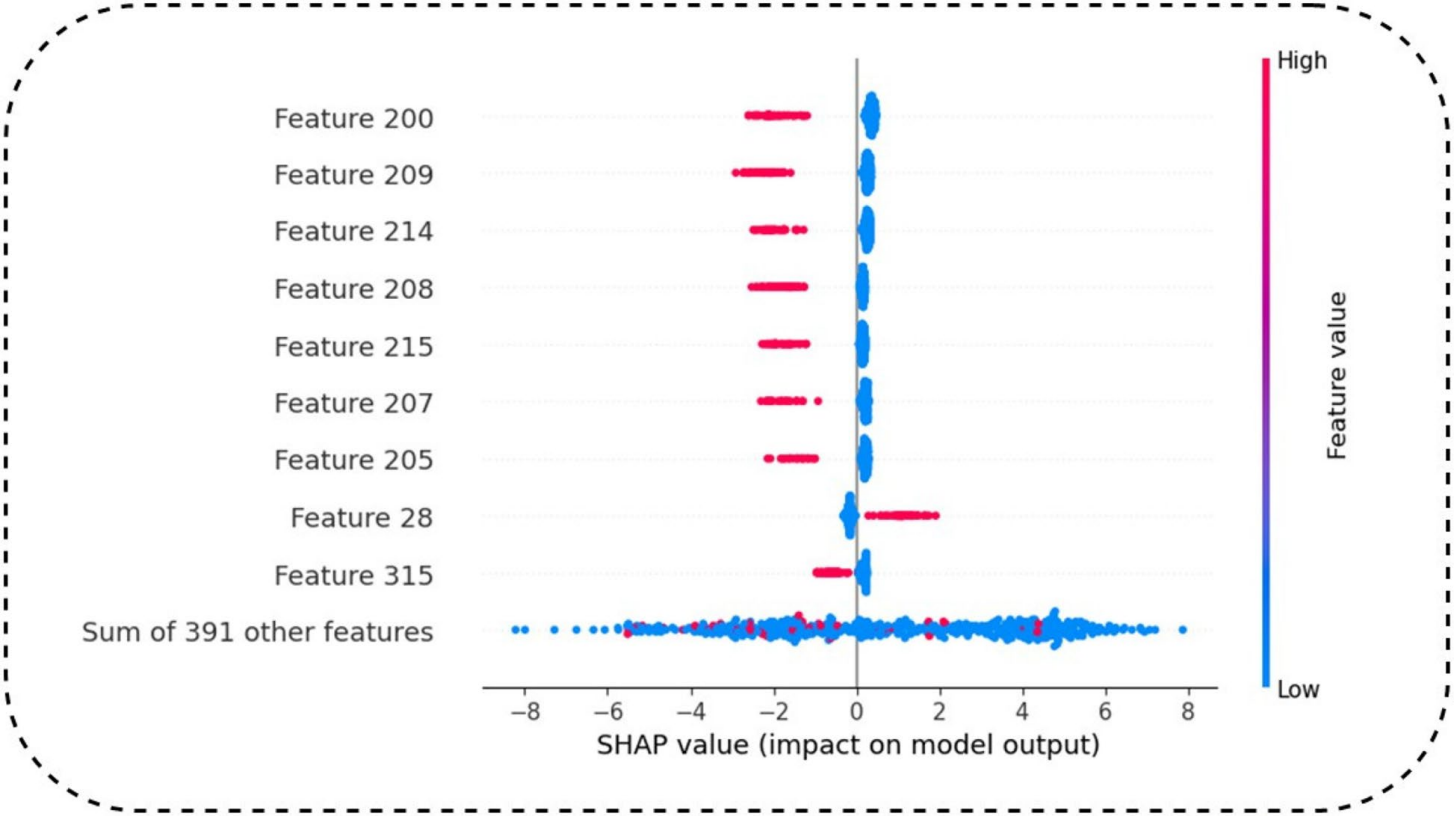
Top 10 features importance based on SHAP feature Analysis

This study's conclusions necessitate more discourse, particularly about the "SHAP" (Shapley Additive exPlanations) technique, which clarifies the influence of each feature on the model's decision-making process as shown in Fig. 11. The models developed with the two different feature combinations were evaluated using MAE and RMSE as outlined in Scikit-Learn. The significant feature values were evaluated using two methodologies: SHAP permutation feature importance and sample visualization by T-SNE. The above graphic is a SHAP (SHapley Additive exPlanations) summary plot, which explains how a few features affect the predictions of a machine learning model. SHAP values (influence on model output) (↔) A positive SHAP value indicates that the characteristic increases the probability of the positive prediction (AMP recognition for instance). Negative SHAP values indicate the feature reduces the risk of the positive class prediction. The Y-axis shows Key Features ranked by importance. Attributes are listed in order of importance. The most important feature is on top. Features 200, 209, and 214 are important due to the fact that the SHAP values of these features vary wildly. SHAP values are not close to zero, indicating that Feature 315 and Feature 28 do not significantly affect the model prediction. Each point represents a SHAP value for a specific feature in an individual prediction. The spread of points laterally indicates how much a feature influences the model for multiple samples. The model output is pushed towards the AMP class by features 200, 209, and 214 (shown in red with high values). Blue (low value) features have a negative effect on the model output. SHAP values, rooted in coalitional game theory, explain each feature's contribution to predictions. Features with a large SHAP range are pivotal for predictions. SHAP values can be positive or negative, reflecting whether a feature supports or contradicts an AMP classification. Color gradient helps to assess if high or low values of a feature are more relevant to predictions. Few factors are importantˣ, so your model relies on certain biological markers to predict AMP. Karhunen–Loeve Transform: it is used to transform a set of vectors into separate uncorrelated values with decreasing information when proceeds (they offer less importance), so it can improve feature selection by removing less important traits improving efficiency. This interpretability aspect of SHAP turns it into an even more reliable model, especially in biological applications where understanding feature impact on outcome is crucial. We have seen that 12 and 4 features associated with features 17 and 19 respectively211215 significantly improved AMP-CapsNet performance. This implies that the feature fusion approach played a vital role in improving the predictive capability of the AMP-CapsNet model.

### Samples visualizationvia* T-SNE*

**Fig. 12:**
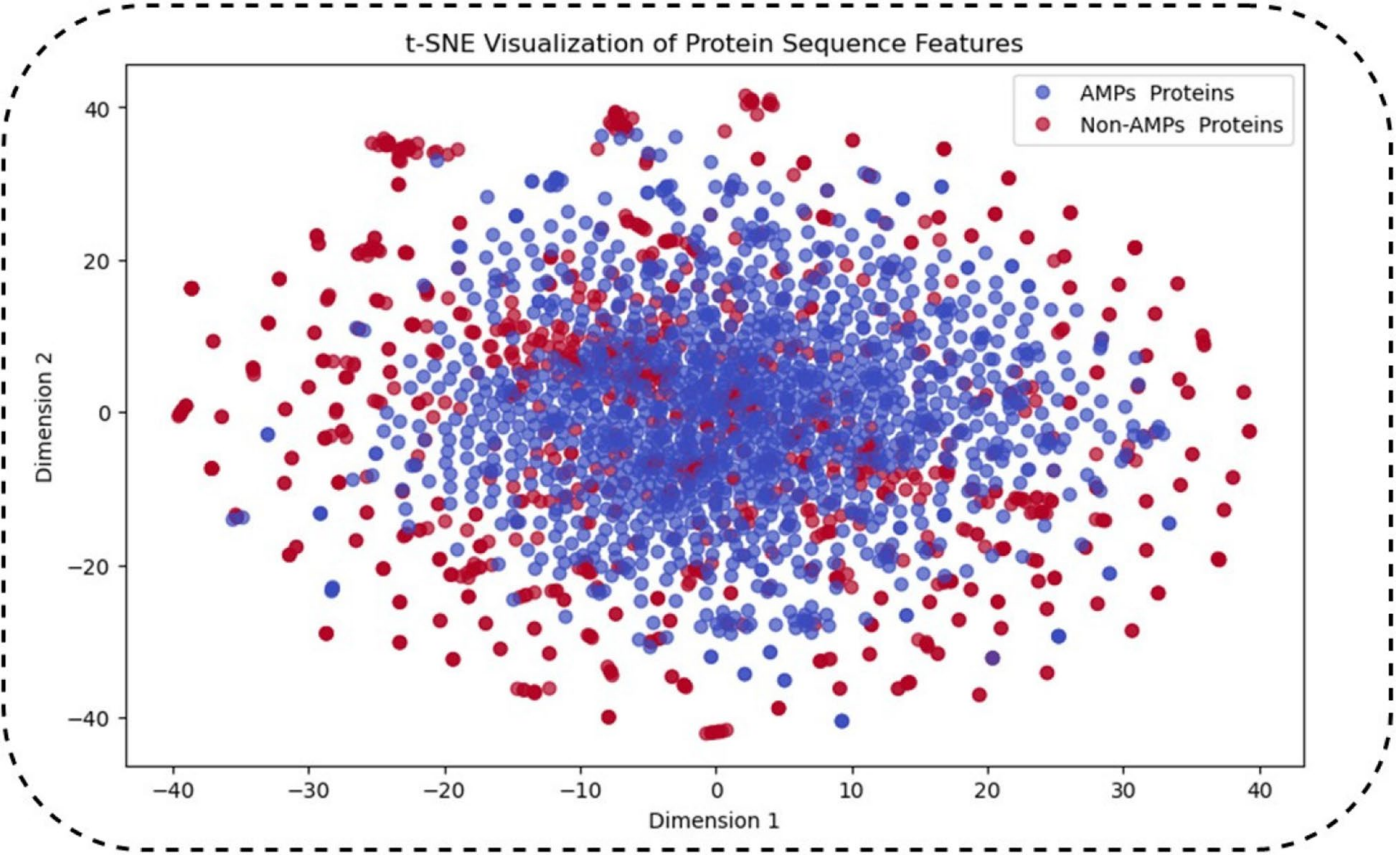
Features visualization based on t-SNE Analysis

To analyse the effect of feature selection on AMP sites prediction we employed t-distributed stochastic neighbor embedding (t-SNE) method. This analysis used t-SNE from the scikit-learn package. The t-SNE method with n-components = 2, perplexity = 50, and learning-rate = 1000 was also applied to the original data (Fig. 12). The present results correspond with the Testing dipeptide composition (DPC). method training set, which includes 400 dimensions, as shown in Fig. 12.

Graphical overview of t-Distributed Stochastic Neighbor Embedding (t-SNE) representation of protein sequence characteristics of AMPs versus non-AMPs. t-distributed Stochastic Neighbor Embedding (t-SNE) is a non-linear dimensionality reduction technique used for embedding high dimensional data in a two-dimensional space while preserving as much of the local structure as possible. Title: t-SNE Visualization of Protein Sequence Features; X-axis: Dimension 1; Y-axis: Dimension 2 (compressed feature space) Blue dots: Indicate AMPs (Antimicrobial Peptides) Red dots: Non-AMPs (Non-Antimicrobial Peptides).

Train yourself on data until October 2023 Projection into low-dimensional (specifically 2D) space, where the local structure is conserved as far as possible. Using probabilistic similarity metrics to ensure that similar points in high-dimensional space are close to each other in the 2D encoding. As there is considerable overlap between AMPs and non-AMPs, feature engineering, as well as advanced classification methods, such as deep learning and ensemble learning, can be implemented.

Multiple clusters (if present) could be evaluated by a feature selection algorithm (like a SHAP analysis) to determine the most critical discriminative features. Non-linear correlations suggests deep learning models (CapsNet, CNN, LSTM) might work better than traditional machine learning models. The visualization of feature space via t-SNE shows that AMPs and non-AMPs are separable to some extent. The overlap indicates that AMP prediction is not a simple problem and requires advanced information extraction and classification techniques. To get a high classification accuracy, sophisticated models (i.e.: CapsNet, CNNs, or mixed DL approaches) might be a necessity.

### Comparison using the validation set

The conclusion of this analysis is consistent with earlier studies which also showed that validation set is used for hyperparameter tuning and results in better understand of model performance. Our proposed model however outperforms the other models across most evaluation metrics as is evident in Table 5, which is an interesting side finding. In particular, we achieved a sensitivity of 96.80%, accuracy of 97.29% and MCC of 94.54% for the validation set. A possible interpretation of this finding is that SeqGAN demonstrates superior performance in precision and specificity, while our model achieves comparable results, exhibiting accuracy values that are 11.98% lower than those of Amplify respectively. Conversely, our model achieves superior performance overall, with Sens and MCC values that are 14.94% and 23.75% greater than those of SeqGAN [42], respectively. The Amplify ranks as the second-best performer in and Sensitivity, attaining values that are 14.94% and 18.81% lower than our proposed model. Taken altogether, the data presented here provide evidence that all methods employing conventional approaches demonstrated performance comparable to the DNN-based classifier in terms of Precision and Specificity metrics. This aligns with recent studies indicating that DNN-based models and shallow learning-based models demonstrate comparable performance [43].

**Table 5:** Performance of diverse models with the validation dataset

Testing Datasets					
Algorithms	**Methods**	**Acc (%)**	**Sn (%)**	**Sp (%)**	**MCC**
RF	AmPEPpy	79.99	70.50	89.48	61.09
RF	MACREL	82.35	77.99	86.70	64.94
DNN	AMPScanner2	82.83	78.60	87.06	65.90
DNN	Amplify	84.16	77.99	90.33	68.84
DNN	SeqGAN	85.31	81.86	88.75	70.79
CapsuleNet	CapsuleNet- AAC	84.42	76.20	90.49	68.58
CapsuleNet	**CapsuleNet- DPC**	**97.29**	**96.80**	**97.70**	**94.54**

This study evaluated different machine learning and deep learning algorithms in the classification of antimicrobial peptides (AMPs) using various feature extraction methods. Using the testing dataset, the accuracy (Acc) [44], sensitivity (Sn) [45], specificity (Sp), and Matthews correlation coefficient (MCC) [46] were evaluated from the models. The results highlight differences in categorization efficacy between the different techniques.

### Assessment of random forest (RF) models

We assessed the effectiveness of the Random Forest (RF) classifier with two features extraction methods, AmPEPpy and MACREL. Therefore, the RF model with AmPEPpy achieves 79.99%, 70.50% and 89.48% of the accuracy, sensitivity and specificity respectively. The MCC of 61.09% indicates a moderate correlation between the predicted and actual classifications. Conversely, RF with MACREL showed better overall performance with an 82.35% accuracy, higher sensitivity (77.99%), and higher specificity (86.70%). A better predictive capability of MACREL was revealed by the MCC of 64.94% compared to RF-AmPEPpy, making it a more compact feature representation for RF.

We show that deep learning models, specifically Deep Neural Networks (DNNs) yield superior classification performances compared to Random Forest (RF). The DNN based on AMPScanner2 achieved an accuracy of 82.83%, a sensitivity of 78.60% and a specificity of 87.06%, giving a Matthews correlation coefficient (MCC) score of 65.90%. The DNN-Amplify model improved classification performance with accuracy of 84.16% and higher specificity (90.33%) than AMPScanner2. Additionally, DNN with SeqGAN achieved the highest accuracy 85.31% and the highest sensitivity 81.86% in AMP detection task, which indicated that it possesses strong proficiency in AMP detection. This MCC of 70.79% reveals a relatively more fair and efficient classification compared with other DNN models.

#### CapsuleNet model evaluation

Two different feature extraction methods, namely AAC and DPC, were used to evaluate Capsule Neural Networks (CapsuleNet). As a result, CapsuleNet-AAC achieved an accuracy of 84.42%, which successfully outperforms DNN models and shows an adequate efficacy against this specific task. The best classification performance with an accuracy of 97.29%, sensitivity of 96.80%, and specificity of 97.70% was achieved by CapsuleNet with capsule vectors features DPC features. MCC value adalah 94.54% (hampir sempurna). This result indicates that the combination of CapsuleNet and DPC features enhances the ability to capture temporal correlations and spatial features, leading to better performance compared to other models.

### Results and conclusion of comparative analysis

The results show that deep learning algorithms, specifically CapsuleNet, outperformed traditional machine learning models like Random Forest. Erbal4 Classification performance approaches based on different feature extraction strategies. The accuracy and overall classification performance of CapsuleNet with DPC feature outperforms all other approaches even though DNN models produce promising results. The data suggest that CapsuleNet-DPC is the most effective method for AMP classification and can probably be considered the state-of-the-art in this context.

## Discussion

We would encourage research to examine and focuses on delineating specific approaches for the evaluation of antimicrobial peptides. Our findings are beyond this case study. This ensures a comprehensive and impartial evaluation of the model. We evaluated the efficacy of ensemble learning and deep learning models in predictive tasks by contrasting various assessment metrics. The results demonstrated that our AMP-CapsNet model attained the highest accuracy, nearing 97.29% on the test set, hence highlighting its significant predictive efficacy in various forecasting tasks. Research of well-defined approaches to the evaluation of antimicrobial peptides deserves to be conducted, and we would like to encourage such studies. A comparison of different models: the results prove that our AMP-CapsNet model achieved the highest accuracy at approximately 97.29% on the test set, greatly indicating the predictive power of our models in multiple forecasting scenarios. These observations demonstrate the possible utility of ensemble learning and deep learning strategies for biomolecular investigations. Such algorithms are capable of deciphering complex biological data successfully and the AMP-CapsNet model presents a significant capability for the quick discovery of potent antimicrobial peptides. Although we plan to improve this model adding other features and finetuning hyperparameters to make the model better in predicting. "We are primarily focused on providing effective solutions to combatting antibiotic resistance.

## Conclusion

We would encourage researchers to examine a novel generation of antimicrobials. Antibiotic peptides represent an appropriate strategy to address antibiotic resistance. The experimental identification of antimicrobial peptides is both time-intensive and difficult. We implemented a deep learning methodology known as the peptides that Capsule Neural Network (AMP-CapsNet) to correctly expect them and assessed its accomplishment in comparison to deep learning and baseline models. The data revealed that the AMP-CapsNet deep learning model achieved an accuracy of 97.29% on the testset dataset, with an AUC score approaching 0.9895, underscoring its remarkable predictive efficacy utilizing dipeptide Composition (DPC). Our research offers innovative insights and prospects for the prediction and detection of antimicrobial peptides. In the field of AI: Through the Lens of Antimicrobial Peptide Discovery Peeling Back the Onion: An Overview of Machine Learning in Antimicrobial Peptide Discovery This study investigates the implementation of deep learning methods in developing prediction models for antimicrobial peptide discovery to reach an ideal integration of experimental laboratory approaches with modelling and establishing theoretical frameworks. Antibiotic peptides are a suitable approach to combat antibiotic resistance. Identifying antimicrobial peptides experimentally is time-consuming and challenging. We designed a novel deep learning architecture AMP-CapsNet on top of rigorous parameters tuning and extensive performance assessment. To accurately predict them, we developed an approach using a deep learning method called the peptides that Capsule Neural Network (AMP-CapsNet) and evaluated its performance against established deep learning and baseline models. The results from the 2000 test data indicated that the AMP-CapsNet deep learning model obtained a 97.29% accuracy and an AUC value approaching 0.9895, demonstrating the excellent predictiveness of this model using Dipeptide Composition (DPC). Our study provides novel approaches and opportunities for the prediction and detection of antimicrobial peptides. It highlights the implementation effectiveness of different approaches used in deep learning and ensemble learning to drive vis-à-vis practicality elements as well as resource constraints to upscale versatility, thereby providing opportunities for new research avenues as well as tools for future investigations of antimicrobial peptides.

## Data Availability

The data provide preliminary evidence to suggest that AMP-1085 and non- AMP-1316, now contain peptide sequences. To train the model and validate it internally (using five-fold cross-validation), we used the in our proposed method experimental total 2401 dataset. To test the model's generalizability, we used the 2401 dataset, which was tested externally. After the datasets were finished, we made sure there weren't any duplicate peptide sequences in either dataset. The reliability of training and evaluating models is guaranteed by this method. https://github.com/ali-ghulam/AMP-CapsNet
